# Therapeutic Potential of Flavonoids in Pain and Inflammation: Mechanisms of Action, Pre-Clinical and Clinical Data, and Pharmaceutical Development

**DOI:** 10.3390/molecules25030762

**Published:** 2020-02-10

**Authors:** Camila R. Ferraz, Thacyana T. Carvalho, Marília F. Manchope, Nayara A. Artero, Fernanda S. Rasquel-Oliveira, Victor Fattori, Rubia Casagrande, Waldiceu A. Verri

**Affiliations:** 1 Departament of Pathology, Center of Biological Sciences, Londrina State University, 86057–970 Londrina, Paraná, Brazil; camila_ferraz96@hotmail.com (C.R.F.); thacy_thacy@yahoo.com.br (T.T.C.); marilia_manchope@hotmail.com (M.F.M.); naayanitelli@hotmail.com (N.A.A.); fernandarasquel@gmail.com (F.S.R.-O.); vfattori@outlook.com (V.F.); 2 Departament of Pharmaceutical Sciences, Center of Health Sciences, Londrina State University, 86057–970 Londrina, Paraná, Brazil

**Keywords:** clinical trials, natural products, hyperalgesia, allodynia, analgesia, flavonoid, hypersensitivity, inflammation, cytokines, NF-kB

## Abstract

Pathological pain can be initiated after inflammation and/or peripheral nerve injury. It is a consequence of the pathological functioning of the nervous system rather than only a symptom. In fact, pain is a significant social, health, and economic burden worldwide. Flavonoids are plant derivative compounds easily found in several fruits and vegetables and consumed in the daily food intake. Flavonoids vary in terms of classes, and while structurally unique, they share a basic structure formed by three rings, known as the flavan nucleus. Structural differences can be found in the pattern of substitution in one of these rings. The hydroxyl group (–OH) position in one of the rings determines the mechanisms of action of the flavonoids and reveals a complex multifunctional activity. Flavonoids have been widely used for their antioxidant, analgesic, and anti-inflammatory effects along with safe preclinical and clinical profiles. In this review, we discuss the preclinical and clinical evidence on the analgesic and anti-inflammatory proprieties of flavonoids. We also focus on how the development of formulations containing flavonoids, along with the understanding of their structure-activity relationship, can be harnessed to identify novel flavonoid-based therapies to treat pathological pain and inflammation.

## 1. Introduction

Inflammatory response induced by micro-organisms or tissue damage trigger the release of pathogen-associated molecular patterns (PAMPs) and damage-associated molecular patterns (DAMPs), respectively [1,2]. Tissue-resident immune cells such as macrophages and dendritic cells (DC) recognize these molecules though receptors namely pattern recognition receptors (PRRs). Once activated, these immune cells produce chemoattractant molecules, which are mainly governed by the transcription factor NF-κB [1,2]. The transcription factor NF-κB regulates the expression of inflammatory enzymes, such as COX-2 [3] and pro-inflammatory cytokines [4,5,6], which makes it one of the most important transcription factors during the inflammatory process and pain. Cytokines and chemokines released by these immune cells along with formyl-peptide (fMLP) released by dying cells activate vascular endothelial cells and provide a gradient of signals that precisely guide neutrophils to the inflamed tissue following a spatial, temporal and hierarchic cascade of mediators [7,8]. Specifically, neutrophils rapidly migrated away from high concentrations of CXCR2 ligands to follow fMLP signal, indicating that the necrotactic stimulus hierarchically override CXCR2 signaling. Accordingly, the lack of fMLP receptor, but not CXCR2, impairs the chemotaxis of neutrophils to the necrotic foci in the context of sterile inflammation [8]. In addition to follow a spatial, temporal and hierarchic cascade of mediators, the recruitment of neutrophils is also context dependent. Using *E. coli* as stimulus, it has been demonstrated that the adhesion of neutrophils in sinusoids depends on CD44 rather than Mac-1 (required for sterile inflammation), revealing, therefore, different adhesion molecules for neutrophil recruitment in sterile vs non-sterile inflammation [8]. In the inflammatory foci, neutrophils release reactive oxygen species (ROS), pro-inflammatory cytokines, and pro-inflammatory lipid mediators, which ultimately contribute to inflammatory pain. Prostaglandin (PG) E_2_, for instance, is produced by either COX-1 or COX-2 and during inflammation, increased production of PGE_2_ by COX-2 contributes to neuronal sensitization leading to pain [9]. Nociceptive pain has a protective function and occurs upon the detection of noxious stimuli by nociceptor. For the detection of these stimuli, nociceptors express ion channels such as the TRP (TRPA1, TRPM8, TRPV1 etc), Nav (Nav1.7, Nav1.8, Nav1.9 etc), and ASIC (ASIC1 to 4) families in their peripheral ends [9]. Pathological pain, on the other hand, is characterized by an amplified response to a noxious stimulus (hyperalgesia) or by a response to a normally innocuous stimulus (allodynia) [10]. Nociceptive pain, therefore, involves the activation of high threshold nociceptors while in inflammatory pain, non-noxious stimuli can now generate mechanical and thermal hypersensitivity [9,11,12]. This phenomenon is described as nociceptor sensitization and occurs due to a shift from a high threshold to a low threshold type of pain [9,11,12]. Current knowledge on persistent pathological pain includes the understanding on how peripheral and spinal cord sensitization of nociceptors occur and changes in the immune cell phenotypes influences pain perception. In the periphery, innate immune cells such as macrophages, neutrophils, and mast cell release mediators that act on the peripheral nerve endings [9,11,12]. PGE_2_, histamine, and cytokines, for example, are the main molecules responsible for lowering neuronal activation threshold and producing peripheral neuronal sensitization [9,11,12]. In the spinal cord, this sensitization is mediated by cytokines, chemokines, and growth factors released by tissue resident cells known as microglia, astrocytes, and oligodendrocytes [13,14,15,16]. All of these mechanisms ultimately contribute to pathological pain by promoting plastic changes in the periphery and central nervous system (CNS) that modify the neuronal phenotype and function.

Pain management is a worldwide challenge due to side effects induced by classical treatments. Acetaminophen and NSAIDs are effective for the management pain. While preclinical data demonstrate that COX-2 selective inhibitors are effective, clinical data show that they induce several side effects such as kidney and heart diseases [17], and non-selective COX inhibitors also induce gastro-intestinal ulcers and kidney injury [18,19]. Acetaminophen is widely known to induce liver injury both in mouse and human [20,21]. This means that there is need of drugs with lessened side effects or different side effects allowing to choose the best option considering the patient’s comorbidities. Depending on the intensity of the pain, opioids are one of the drugs used for relief. However, millions of patients cope with side effects that include constipation, drowsiness, risk of addiction, and sometimes even respiratory failure and death [22]. Even upon opioid therapy, neuropathic pain, for instance, remains challenging to treat, with only half of the treated population typically report a significant reduction in pain and complete resolution of symptoms is rarely achieved [23]. Paradoxically, while recognized by their potent analgesic effects, opioids can induce pain [24]. The release of DAMPs, such as HMGB-1 and biglycan, induced by morphine is of the main mechanisms related to opioid-induced pain [25]. These molecules increase the production of spinal cord IL-1β by microglia in a NLRP3-dependent manner [26]. Furthermore, morphine increases IL-1β mRNA expression via TLR4 and increases IL-1β release via P2X4 receptor [27]. Altogether, this cascade of events in microglia potentiates morphine-induced hyperalgesia by stimulating IL-1β production in the spinal cord and explains this paradoxical effect [25,26,27]. Corticosteroids and immunobiological agents are other group of molecules used to treat pain. While the first cause hormonal changes (leading to suppression of adrenal function and bone loss) and Cushing syndrome [28], the later induces heart failure [29]. Moreover, corticosteroids, immunobiological agents, and opioids are linked with immunosuppression and consequently pathogen spread [30,31,32]. Thus, the search for natural compounds with lower adverse effects on patients has been growing, and over the past years higher attention has been given to the flavonoids.

**Figure 1:**
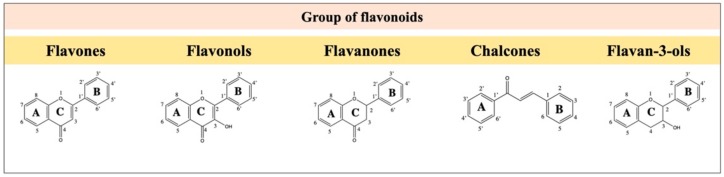
The chemical structures of the flavonoid groups discussed in this review.

Flavonoids are an essential group of polyphenolic compounds, and their flavan nucleus is the main structural characteristic. Figure 1 shows the structures of the flavonoids discussed in this review.

They are one of the most widely found classes of compounds in vegetables and fruits. The chemical structure of flavonoids is based on a fifteen-carbon skeleton consisting of two benzene rings connected through a heterocyclic pyrane ring. The flavonoids can be divided into an assortment of classes, for example, flavones (e.g., flavone, apigenin, and luteolin), flavonols (e.g., quercetin, kaempferol, myricetin, and fisetin), and flavanones (e.g., flavanone, hesperetin, and naringenin). While individual compounds within a class differ in the pattern of substitution of the A and B rings, the classes itself vary in the degree of oxidation and pattern of substitution of the C ring. Flavonoids are known to have analgesic, anti-inflammatory, and antioxidant properties. These effects are related to the inhibition of NF-κB-dependent pro-inflammatory cytokines [33], VEGF, ICAM-1, STAT3 [34], and activation of antioxidant transcription factor Nrf2 [33]. Many flavonoids, as apigenin and vitexin, were reported to be safe natural alternative therapeutic against neuroinflammatory diseases as such Multiple Sclerosis (MS) [35,36]. Therefore, flavonoids are multi-target drugs, which explain their wide array of actions. Further, as the activity of flavonoids does not depend on abolishing a single mechanism, but rather reducing varied mechanisms, it is likely that physiological function is maintained, which reduces the incidence of side effects compared to single target drugs.

## 2. Pre-Clinical Evidence of Flavonoids for Pain Control

**Figure 2:**
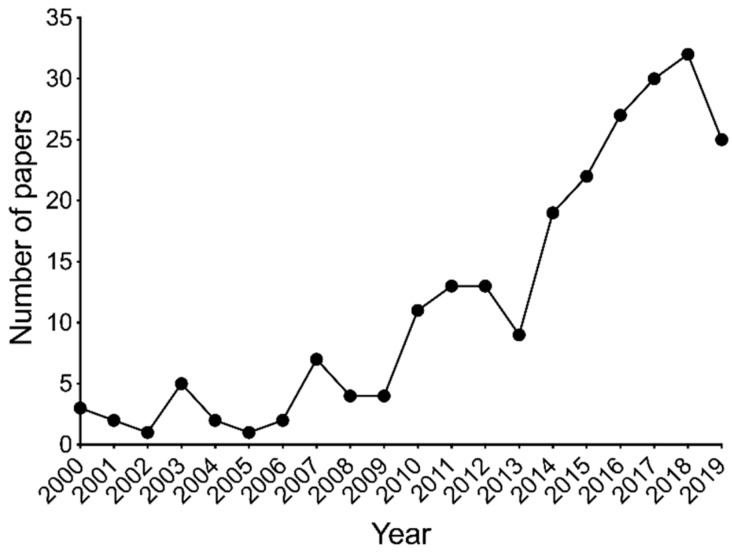
The number of manuscripts published on flavonoids, pain, and inflammation during the last 20 years at PubMed. The keywords search at PubMed was “flavonoids and pain and inflammation”, and only original research papers were considered.

Many mediators are produced by tissue immune resident cells and immune cells that undergo recruitment to the injured tissue during an inflammatory response. Among these mediators, PGE2 [37], along with bradykinin, sympathetic amines, and pro-inflammatory cytokines (e.g., TNF-α, IL-6, and IL-1β) act to promote either sensitization or activation of nociceptors and then evoke pain [37,38]. Flavonoids are plant derivative compounds, and their potential usage as medicinal natural products is growing exponentially over the years (Figure 2).

**Figure 3:**
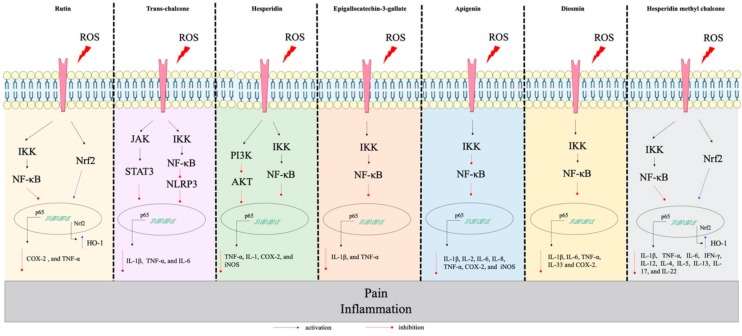
The anti-inflammatory and analgesic effects of flavonoids. Intracellular targets of **Rutin**: NF-κB [39,40] and Nrf2 [40], **Trans-chalcone**: NF-κB [41] and STAT3 [41] and NLRP3 [42], **Hesperidin:** PI3K/ AKT [43] and NF-κB [44], **Epigallocatechin-3-gallate:** NF-κB [45], **Apigerin**: NF-κB [46], **Diosmin**: NF-κB [47], and **Hesperidin methyl chalcone:** NF-κB [48,49,50] and Nrf2 [49,50]. ROS and inflammatory stimuli that activate specific receptors trigger intracellular signaling that will result in pain and inflammation. The **blue** arrows indicate endogenous pathways that are stimulated by flavonoids resulting in the reduction of pain and inflammation. The **red** arrows represent endogenous pathways that are inhibited by flavonoids resulting in reduced pain and inflammation.

**Figure 4:**
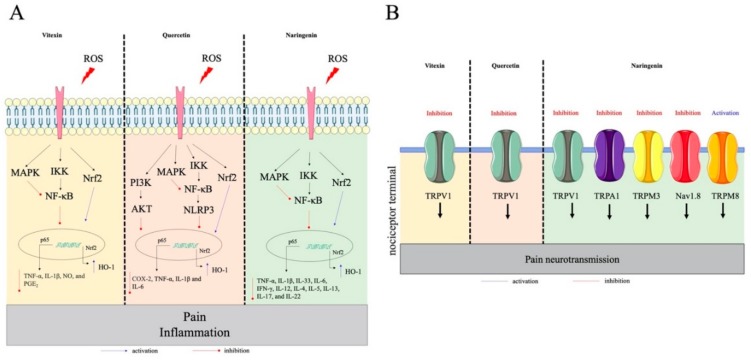
The anti-inflammatory and analgesic effects of Vitexin, Quercetin, and Naringenin. (**A**) Intracellular targets of **Vitexin**: MAPK [51], NF-κB [51] and Nrf2 [52], **Quercetin**: MAPK [53], NF-κB [53,54,55], AKT [56], Nrf2 [33,54], and NLRP3 [57] and **Naringenin:** NF-κB [58,59,60] and Nrf2 [59,61,62]. (**B**) Ion channels expressed by neurons that are targeted by Vitexin, Quercetin, and Naringenin to reduce pain. **Vitexin**: TRPV1 [38], **Quercetin**: TRPV1 [63], and **Naringenin:** TRPV1 [58], TRPA1 [58], TRPM3 [64], Nav 1.8 [65], and TRPM8 [64]. In panel (**A**), ROS and inflammatory stimuli that activate specific receptors trigger intracellular signaling that will result in pain and inflammation. The **blue** arrows indicate endogenous pathways that are stimulated by flavonoids, and the **red** arrows represent endogenous pathways that are inhibited by flavonoids resulting in reduced pain and inflammation.

Studies about flavonoids’ effects on inflammatory diseases and pain have been increasing in the last decade as several groups are demonstrating the involvement of these phenolic compounds as anti-inflammatory, analgesic, and antioxidant molecules. The search for new therapeutic drugs with less or no side effects is the major reason leading to this growing interest in natural products for the treatment of inflammatory and painful conditions. The flavonoids discussed in this section were selected based on the ones that also present clinical data (Section 4: Clinical Studies and Safety), to align pre-clinical data with clinical data. We discuss their targets in the disease context, which explains the anti-inflammatory and analgesic actions of flavonoids (Figure 3 and Figure 4). Figure 3 shows flavonoids whose anti-inflammatory and analgesic effects do not involve, at least at the present date, the direct interaction and modulation of ion channels.

### 2.1. Flavonols

Quercetin (3,3′,4′,5,7-pentahydroxyflavone) is a flavonol found in different types of fruits, vegetables, and plants, including: berries, apples, tomatoes, cocoa, onions, and medicinal plants [66,67,68]. It is an antioxidant flavonoid that presents anti-inflammatory activity by inhibiting pro-inflammatory cytokines in mouse models of colitis [69], periodontitis [70], cancer pain [71], and acute [54] and chronic arthritis [33]. That effect is mainly related to the ability of quercetin in reducing recruitment of neutrophils (myeloperoxidase [MPO] activity [33,69,72]), oxidative stress [33,54,69,73,74], COX-2 in the knee joint (arthritis model) [33], NLRP3 inflammasome activation (inhibition of ASC speck formation and ASC oligomerization) in macrophages [57], p65 NF-κB activation [53,54] and MAP kinases signaling in macrophages [53], p50 NF-κB activation in primary human keratinocytes [55], and TNF-α, IL-1β and IL-6 production in RAW 264.7 cells stimulated by LPS [75]. Recent studies demonstrated that quercetin is able to modulate the neutrophils actin polymerization [76], pro-inflammatory cytokines expression by mast cells [77,78] and monocytes [79,80], dendritic cell activation [81] and maturation [82], and the phenotype M1 to M2 in macrophages [83,84,85,86]. In the past few years, increasing attention has been paid to the analgesic effect of quercetin [33,54,71,73,74,87]. In addition to the inhibition of the aforementioned pro-inflammatory signaling pathways, part of that analgesic effect is through the activation of Nrf2/HO-1 pathway [33,54]. In fact, co-treatment with a HO-1 inducer potentiates the analgesic effect of opioids and cannabinoids through the activation of cGMP/PKG/ATP-sensitive potassium channels pathway in a model of CFA-induced pain [88], indicating that stimulating HO-1 expression contributes to analgesia. This is a relevant mechanism because it has been demonstrated that morphine activates PI3Kγ/AKT pathway that, in turn, stimulates NO production in an nNOS-dependent manner in the DRG neurons [89,90]. NO also indirectly acts through stimulation of cGMP/PKG and causes the up-regulation of ATP-sensitive potassium channels to promote the hyperpolarization of primary nociceptive neurons [89,90]. Moreover, when phosphorylated, p65 subunit competes with Nrf2 for the adaptor protein CREB binding protein (CBP) [91,92]. Consequently, there is a hypoacetylation that blocks chromatin condensation, suppressing Nrf2/ARE gene expression. That mechanism dampens the Nrf2 signalling pathway activation and consequently antioxidant response [91,92]. Quercetin can also suppress the expression levels of PKCε and TRPV1 in the spinal cords and DRGs as demonstrated in a model of paclitaxel-induced peripheral neuropathy in rats and mice [63]. Therefore, drugs such as quercetin with the ability of activating cGMP/PKG/ATP-sensitive potassium channels pathway in neurons without the important side effects of opioids might be promising candidates for pain treatment.

Rutin (3,3′,4′,5,7-pentahydroxyflavone-3-rhamnoglucoside), a glycoside of quercetin, is also a flavonol that can be found in several plants, such as buckwheat, white mulberry, passion flower, tomatoes and apples [93,94]. It possesses anti-inflammatory and anti-hyperalgesic effects through the inhibition of oxidative stress and neuroinflammation [39]. Specifically, rutin inhibits NF-κB activation [39,40], activates Nrf2/HO-1 and NO/cGMP/PKG/ATP-sensitive potassium channels channel signaling pathways and inhibits pro-inflammatory cytokine production (IL-1β and TNF-α) [40]. Rutin promotes M2 polarization of Th1 primed RAW264.7 and CD11b+ primary macrophages [95] and modulates the production of mediators by neutrophils [96], mast cells [77], and monocytes [97]. Furthermore, by testing the same doses of rutin and quercetin (80 mg/kg), it was demonstrated that rutin was more efficient than quercetin on reducing edema in a model of experimental arthritis of adjuvant-carrageenan-induced inflammation (ACII) in rats likely because better absorption due to higher hydrophilicity than quercetin [98]. Altogether, these data indicate that both quercetin and rutin block pain and inflammation by activating cGMP/PKG/ATP-sensitive potassium channels pathway in neurons and inhibiting NF-κB and inducing Nrf2 activation in immune cells.

### 2.2. Flavones

Apigenin (4′,5,6-trihydroxyflavone) is abundantly present in vegetables, fruits, and medicinal herbs, including parsley, apples, grape, and *Matricaria chamomilla* [99,100]. It was demonstrated that this flavone possesses anti-inflammatory effects by blocking NF-κB activation in a model of LPS-induced apoptosis. In this model, apigenin decreases the neutrophil infiltration to the lungs by reducing the Macrophage Inflammatory Protein-2 (MIP-2) that is a neutrophil chemoattractant molecule [46]. Furthermore, previous studies showed apigenin has anti-inflammatory activities on LPS-induced inflammation through the inhibition of the expression of iNOS, COX-2, cytokines (IL-1β, IL-2, IL-6, IL-8, and TNF-α), and AP-1 proteins in human lung epithelial cells [101]. In addition, apigenin modulates macrophages polarization [102], promotes down-regulation of Mcl-1 in neutrophils [103], suppresses the inflammatory activity of DCs [35,104], regulates the production of inflammatory mediators by monocytes [105] and mast cells [106]. Among the antioxidant effects, apigenin scavenges hydroxyl (^•^OH) radicals generated by UV photolysis of hydrogen peroxide [107] and chelates iron [108]. Further, apigenin reduces the levels of MPO and malondialdehyde activity (MDA) in acetic acid-induced ulcerative colitis and these effects were comparable to the corticoid, prednisolone [109].

Vitexin, a flavone C-glycoside (5,7,4-trihydroxyflavone-8-glucoside) found in medicinal and other plants, presents anti-inflammatory activities by preventing the oxidative stress, inhibiting pro-inflammatory cytokines production, and increasing the anti-inflammatory cytokine IL-10 [38]. Vitexin inhibited the production of TNF-α, IL-6, nitric oxide (NO), and PGE_2_ in human osteoarthritis chondrocytes [110]. Furthermore, vitexin prevents mast cells degranulation [111], reduces the production of pro-inflammatory mediators by neutrophils [112,113] and macrophages [113]. Vitexin attenuated RANKL-induced activation of MAPK signal pathways and NF-κB signaling pathways [51]. Also, the activation of Nrf2/HO-1 pathway is one of the mechanisms of vitexin to limit inflammation in a model of LPS-induced acute lung injury [52]. It was demonstrated by Chen et al., [114] in isoflurane-treated PC12 cells that vitexin is neuroprotective by downregulating the expression of TRPV1 and glutamate ionotropic receptor NMDA type subunit 2B (NR2B) protein expression. In corroboration, in vivo data show that vitexin reduces capsaicin-induced pain behaviors, indicating that part of its analgesic effect is through inhibition of TRPV1 activation [38]. Moreover, vitexin showed analgesic activities by reducing the writhing response induced by acetic acid [38,115] and phenyl-*P*-benzoquinone (PBQ) [38], and the thermal and mechanical hyperalgesia [38,115]. The analgesic effects of vitexin in the model of hind-paw incisional surgery seem to be mediated by opioid-related mechanisms since delta, mu, and κ-opioid receptor antagonists reversed the analgesic effects of this flavonoid [115].

Diosmin (diosmetin-7-*O*-rutinoside) is a flavonoid abundant in citrus fruits [116]. This flavonoid presents anti-inflammatory and anti-hyperalgesic mechanisms described in different models. Some of these effects have been attributed to the reduction of NF-κB activation [47], TNF-α in PC12 cell induced by LPS [117], *Il-1β, Tnf-α, and Il-33/St2* mRNA expression in the spinal cord induced by CCI [118], TNF-α, IL-1β and IL-6 in the spinal cord and sciatic nerve tissues [119], NO, PGE_2_, IL-6, IL-12, TNF-α production by macrophages [120], COX-2 and MPO activity [121,122]. In addition, diosmin inhibited LTB_4_ synthesis [121]. The antioxidant effects of diosmin were demonstrated in colitis induced by acetic acid or trinitrobenzenesulfonic acid (TNBS). Diosmin reduced the levels of MDA and prevented the consumption of GSH [121,122]. Regarding the analgesic effects of diosmin, this flavonoid reduces neuropathic pain in the sciatic nerve chronic constriction injury (CCI) model by activating the analgesic NO/cGMP/PKG/ATP-sensitive potassium channels channel signaling pathway and blocking central sensitization [118]. Also, in a model of CCI in rats, diosmin acts at central level through opioid and dopaminergic receptors to inhibit mechanical and thermal hyperalgesia [119]. Unpublished data of the Verri laboratory also show that diosmin treats LPS-induced peritonitis and inflammatory pain by blocking NF-κB activation in leukocytes. Therefore, diosmin might be a promising drug to treat chronic and non-sterile inflammatory pain.

### 2.3. Flavanones

Naringenin (4′,5,7-trihydroxyflavanone) is found in citric fruits such as lemon, grapefruit, orange, and tangerine [123]. Its effects on inflammation have been demonstrated in UVB irradiation [61,124], inflammatory pain [58,62,125], and arthritis [59]. The mechanisms by which naringenin reduces inflammation and pain are related to inhibition of oxidative stress [61,62,124,126], leukocyte recruitment [59,60], MPO activity [124,127], NF-κB activation [58,59,60], mRNA expression of inflammasome components [59], pro-inflammatory cytokine production (TNF-α, IL-1β, IL-33, IL-6, IFN-γ, IL-12, IL-4, IL-5, IL-13, IL-17, and IL-22) [58,59,60,62,124,125,127], and MAPK signaling activation [128]. In addition, naringenin modulates macrophages activation [129] and microbicidal activity of neutrophils [130], and reduces DC maturation [131]. Further, naringenin acts by the inducing Nrf2/HO-1 [59,61,62] and activation of the analgesic signaling pathway NO/cGMP/PKG/ATP-sensitive potassium channels [62]. Naringenin also reduces formalin- and capsaicin-induced pain behaviors, demonstrating that part of its mechanism is through inhibition of TRPA1 and TRPV1 activation, respectively [58]. In addition, naringenin induces analgesia by blocking TRPM3 and activating TRPM8 [64], which might contribute to its analgesic effect. Naringenin also blocks sodium influx in rat DRG neurons by selecting inhibiting Nav1.8 (and not Nav1.7 or Nav1.9) [65]. Collectively, naringenin targets different channels expressed by neurons and immune cell signaling pathways (decreases NFkB activation and stimulates Nrf2) to reduce pain and inflammation.

Hesperidin (hesperetin-7-rhamnoglucoside) is a flavonoid that belongs to the flavanones class mainly found in citrus fruits [132]. Pharmacological effects of hesperidin have been reported such as anti-inflammatory [133,134], analgesic [134,135,136], antioxidant [133,137], antiallergic [138], and antidiabetic [139]. Hesperidin reduces TNF-α, IL-1β, ICAM-1, VEGF, the neutrophil ability to generate superoxide radical [140], and advanced glycosylation end products levels [133]. Also, hesperidin modulates polarization of macrophages to M1 through suppression of the PI3K/AKT pathway [43], and inhibits TNF-α and IL-1β production by mast cells [141]. Further, it was demonstrated that hesperidin reduces MDA levels [44,133] and increases the antioxidant enzyme superoxide dismutase (SOD) activity [133] and GSH [44]. Moreover, hesperidin inhibits neuroinflammation in mice as observed by decreased levels of GFAP (an astrocyte activation marker), NF-κB, iNOS, and COX-2 in the coronal brain induced by an intracerebroventricular infusion of streptozotocin (STZ) [44]. Treatment with hesperidin also ameliorates mechanical hyperalgesia in STZ-induced diabetic rats [136] and CCI experimental model of neuropathic pain [134]. Therefore, both hesperidin and naringenin show neuroprotective and antioxidant properties that contribute to their analgesic effects.

### 2.4. Chalcone

Under alkaline conditions, the methylation of hesperidin produces hesperidin methyl chalcone (HMC) [142]. HMC is an antioxidant flavonoid with anti-inflammatory and analgesic effects in experimental models. For instance, HMC inhibits oxidative stress by preventing the decrease of the antioxidant capacity (FRAP, ABTS, and GSH) and inhibiting the superoxide anion production, lipid peroxidation and gp91phox mRNA expression. Inducing the Nrf2/HO-1 pathway [49,50] contributes to the in vivo antioxidant effects of HMC [49,50,143,144]. HMC also inhibits the production of pro-inflammatory cytokines, such as TNF-α, IL-1β, and IL-6 induced by UVB irradiation in mouse skin [50]. Further, HMC inhibited edema, neutrophil recruitment (MPO activity), and MMP-9 activity [143]. HMC showed anti-inflammatory effects by inhibiting the NF-κB-dependent pro-inflammatory cytokine production (IL-1β, TNF-α, IL-6, IFN-γ, IL-12, IL-4, IL-5, IL-13, IL-17, IL-22) [49,50,143,144] and leukocyte recruitment [49,143]. In addition, targeting the TRPV1 receptor activation is another important mechanism by which hesperidin exerts its analgesic effects [144]. We recently demonstrated that HMC interacts with NFκB (Ser276 residue), reduces *gp91phox* and increases *Ho-1* mRNA expression in knee joint tissue after stimulus with zymosan. HMC also reduces the cytokines IL-33, TNF-α, and IL-6 levels in zymosan-stimulated RAW264.7 macrophages [48].

Trans-chalcone (1,3-diphenyl-2-propen-1-one) is a flavonoid precursor that can be obtained from plants such as *Piper methysticum* (Kava-Kava), *Aniba riparia* and *Didymocarpus corchorijolia* [145]. Trans-chalcone presents in vivo antioxidant effects [42,145] and anti-hyperalgesic mechanisms through the inhibition of leukocyte recruitment, IL-1β, TNF-α, and IL-6 production, and NLRP3 inflammasome activation in macrophages stimulated with MSU crystals [42]. It also inhibits VEGF and ICAM-1 expression and activation of STAT3 and NF-κB [41], indicating that part of its analgesic mechanism is related to the blockade of NF-κB-dependent pro-inflammatory mediators.

### 2.5. Flavanols, Flavan-3-ols or Catechins

Epigallocatechin-3-gallate (EGCG) is a polyphenol very abundant in green tea (*Camellia sinensis*) and its main active component [146]. This flavonoid has several biological activities, including anti-inflammatory, anti-hyperalgesic, and antioxidant. EGCG inhibited the COX-2 mRNA expression and PGE_2_ production induced by IL-1β in human osteoarthritis chondrocytes via up-regulation of hsa-miR-199a-3p expression [146]. Another mechanism by which EGCG inhibits inflammation is via down-regulation of NF-κB and consequently inhibition of iNOS stimulated by LPS in mouse macrophages [45], and reduced NO, prostaglandin PGE_2_ and COX-2 production in macrophages [147], dendritic cell differentiation and maturation [148], and inhibited mast cell degranulation [149] and neutrophil chemotaxis [150]. Peripheral analgesic effects of EGCG are related to the suppression of CCR2, IL-1β, and TNF-α mRNA expression in the DRG in a model of OA [151] and through down-regulation of CX3CL1 protein in the spinal cord in a CCI-induced neuropathic pain [146]. In terms of spinal effects, EGCG decreases TNF-α expression in the spinal cord in mouse models of bone cancer-caused pain [152] and reduces glial cell activation via inhibition of fatty acid synthase (FASN), Ras homologue gene family member A (RhoA), and TNF-α in a model of spinal cord injury (SCI) [153].

Summing up, the findings from these studies suggest that treatment with flavonoids effectively alleviate inflammation and pain in varied preclinical models by inhibiting leukocyte recruitment, pro-inflammatory/pro-hyperalgesic mediator production, oxidative stress, activation of signaling pathways (Figure 3 and Figure 4) and the modulation of channels to inhibit inflammation and pain (Figure 4). The data in Table 1 summarize some effects of flavonoids on different cell lines.

## 3. Structure-Activity Relationship (SAR)

**Figure 5:**
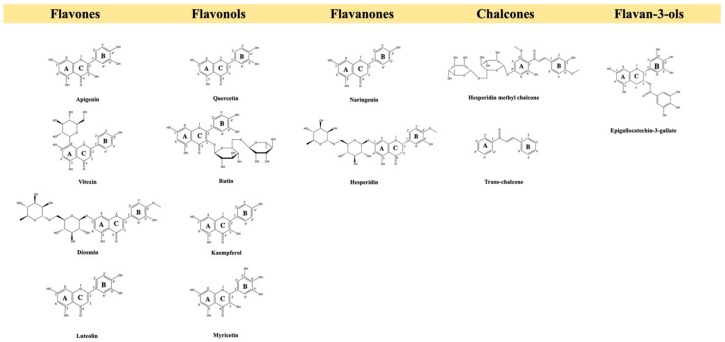
The chemical structures of the flavonoids discussed in this review.

Flavonoids are divided into different classes with a basic structure of 3 rings. Figure 5 shows the basic structure of the flavonoids discussed in this section.

These classes differ on where the C-ring carbon is attached to the B ring, and the C-ring saturation and oxidation degree [154]. Therefore, the structure of the flavonoids is fundamental to the understanding of their activity. Relevant to flavonoids’ biological effect are: (i) B ring containing *O*-dihydroxy confers stability after hydrogen donation, and phenoxyl radical formation which participates in the electron delocalization; (ii) C ring allows electron dislocation from phenoxyl radical from B ring when a 2,3-double-bound bond is in conjugation with 4-oxo group on C ring; (iii) electron dislocation is favorable in combination of 2,3-double bond and 3-hydroxyl and 5-hydroxyl groups by increasing the resonance stability [155]. The amount of hydroxyl groups is less important than their position at flavonoid basic structure. For example, the isovitexin has 3 hydroxyls groups and baicalin has 2 hydroxyls groups differing into hydroxyls amount, positions and scavenging DPPH activity. Isovitexin (apigenin-6-C-glucoside) does not scavenge DPPH radicals (IC_50_ > 1000 µM) while baicalin (Baicalein 7-O-glucuronide) scavenges DPPH radicals (IC_50_ = 15.5 µM). On the other hand, the monomer epicatechin scavenges DPPH radicals at same level as its dimer Procyanidin B-2 (IC_50_ = 11.7 µM) [156]. While the class of flavonoid is not determinant for their activity (for instance, flavonols [kaempferol, quercetin, and myricetin] and flavones [chrysin, flavone, apigenin, baicalein, baicalin, and luteolin] block TNFα-induced ICAM-1 expression on alveolar epithelial cells A549), the hydroxyls at positions 5 and 7 on the A ring and at position 4 on the B ring are important for activity [157]. Specifically, hydroxyls at position 3 on B ring reduce flavonoid activity and at position 5 position abolish its activity [157]. Thus, changes into basic flavonoid structure could increase, decrease, or even not alter flavonoids antioxidant activity. In addition, flavonoids such as trans-chalcone that does not present antioxidant chemical groups presents anti-inflammatory and analgesic effects in vivo and reduce oxidative stress in vivo likely due to inhibiting inflammation since no antioxidant effect was observed in vitro in cell-free systems [42,158]. Thus, defining whether a flavonoid has therapeutic potential solely by its structure and chemical groups with antioxidant potential is not adequate to take full advantage of plant flavonoids. Further, there is more detailed understanding on the structure activity relationship regarding antioxidant activity without clear conclusions on anti-inflammatory and analgesic mechanisms. In this section, we discuss how flavonoid basic structure and their substitutions correlate with their activity.

Flavonoids can be found on nature as their glycoside or aglycone forms. Flavonoid glycosides present increased solubility and stability in water, which, however, interferes with their activity. Given glycosylation occurs in hydroxyl groups, it changes structural key elements for their radical scavenging activity. Specifically, glycosylation changes the double bond in conjugation with the 4-OXO group in the C-ring at C2, C3 position, the *O*-dihydroxy (catechol group) at the B-ring, and the presence of hydroxyl groups in positions C-3 (C-ring), C-5 and C-7 (A-ring) [159]. For instance, the aglycone quercetin and its glycoside form quercitrin both inhibit leukocyte recruitment and LTB_4_ production in carrageenan-induced pleurisy in rats, but not at the same level. Quercitrin showed lower activity compared to its aglycon form quercetin [160]. Also, aglycones quercetin and hesperetin (75 mg/kg) inhibit carrageenan-induced paw edema in mice but not their glycosides form rutin and hesperidin (150 mg/kg, i.p.) [161]. Regarding glycosylation effect on antioxidant activity, quercetin scavenged more DPPH and peroxynitrite ([ONOO^−^], IC_50_= 5.5±0.1 and 48.8 ±0.5 µM) than rutin (IC_50_= 7.4 ±0.2 and 92.4±1.0 µM) [162]. Thus, while glycosylation increases flavonoid solubility, it decreases anti-inflammatory and antioxidant activities because it changes key features in the flavonoid structure.

ONOO^−^ is produced by the reaction between superoxide anion and nitric oxide. Both mediators are produced at high amounts during inflammatory process by NADPH oxidase and inducible nitric oxide synthase [163]. These mediators induce pain by increasing hyperalgesic mediators through NFkB activation [164], such as TNFα acting via TNFR1 [165] and COX-2/PGE2 axis [74,166], or by directly inducing neuronal depolarization. For the scavenge ability of ONOO^−^ in vitro, 3-hydroxyl moiety at B ring demonstrated to be important for flavonoids. For instance, quercetin a flavonoid that possess the 3-hydroxyl group has a higher scavenger ability (IC_50_ = 0.93 ±0.12 µM) when compared to the flavonols galangin (IC_50_ = 3.37 ±0.99 µM) and kaempferol (IC_50_ = 4.35 ±0.27 µM) which have the group 4-hydroxyl [167]. Similarly, the *O*-dihydroxy (catechol group) is determinant to superoxide anion scavenging activity of flavones and flavanones. For instance, the presence of the following elements increases scavenging activity: no hydroxyl groups at the B-ring, a 4′-hydroxyl substitution, and *O*-dihydroxy (catechol group), where the last feature shows higher antioxidant capacity [168]. Therefore, the hydroxyl group at B ring seems to be important for ONOO^−^ and superoxide anion scavenging activity and its reasonable that this scavenging activity accounts for flavonoids analgesic and anti-inflammatory activity. Flavonoids also present antioxidant effects by indirect mechanisms through activation of Nrf2 signaling pathway, for example [169]. Chalcones are more potent than other types of flavonoids, where the double bond at C2-C3 position of their structure are particularly important for Nrf2 induction. In fact, reduction of that double bond impairs Nrf2 activation. Chemical addition of sugar moiety to the flavonoid basic structure or naturally flavonoid glycosides present less activation of this important signaling pathway [170].

The analgesic and anti-inflammatory activities of flavonoids are related, at least in part, to their NF-κB inhibitory effects [40,54,58,60,62]. Shin et al. screened 30 flavonoid derivatives against their inhibitory activity on TNFα-induced NF-κB activation in HCT116 human colon cancer cells. Moiety 3′,5′-dimethoxy at ring B seems to be important to inhibit TNFα-induced NF-κB activation in vitro [171]. Specifically, compounds 2,3′,5′-Trimethoxychalcone, 3,3′,5′-Trimethoxychalcone, 3,3′,5,5′-Tetramethoxychalcone, and 2-Hydroxy-3′,4,5′-trimethoxychalcone had better inhibitory effect than 3,3′,5-Trimethoxychalcone, 2′,3,5-Trimethoxychalcone and 2-Hydroxy-4,4′-dimethoxychalcone. Compound 3,3′,5,5′-Tetramethoxychalcone showed the best inhibitory activity against TNFα-induced NF-κB activation decreasing IkB phosphorylation at Ser^32^ and RelA phosphorylation at Ser^536^. Thus, 3′,3”,5′,5”-Tetramethoxychalcone seems to interfere with IKK complex responsible for IkB and RelA phosphorylation [172]. Moreover, apigenin, a flavone, interferes with IKK complex by decreasing RelA phosphorylation at Ser^536^ in LPS-induced NF-κB activation in human primary monocytes [172]. Comparing 5-hydroxyl against 5-methoxyl substitution at flavone A-ring, hydroxyl moiety displays higher effect than methoxyl in TNFα-induced NF-κB activation in HCT116 cells [171]. Regarding flavonoids, the inhibition of NF-κB activation in a context of TNF-α-induced ICAM-1 expression by luteolin and apigenin is dependent on the presence of a double bond at position C2-C3 of the C ring with OXO function at position 4, along with the presence of hydroxyl groups at position 4′ of the B ring. In fact, chrysin that lacks hydroxyl group at position 4′ of the B ring has lower inhibitory effect over NF-κB activation when compared to apigenin and luteolin [157]. Thus, there are different features in each ring that alters NF-κB inhibitory activity. (i) At B ring 3′,5′-dimethoxy moiety and hydroxyl group at position 5 in ring A, and (ii) at ring C a double bond at position C2-C3 with OXO function at position 4 and combined with hydroxyl group at position 4′ at ring B increased inhibitory activity in TNFα-induced NF-κB activation. More studies are needed to determine more important SAR for flavonoids’ NF-κB inhibitory activity.

As mentioned, flavonoids are drugs with safe pre-clinical profile without the common side effect of standard NSAIDs. For instance, luteolin inhibits PGI_2_ produced by COX-2 without the usual side effects produced by NSAIDs [173,174]. Ribeiro et al. screened at whole blood flavonoids inhibitory activity against COX-1 and COX-2. A common feature of those flavonoids which inhibited COX-2 activity (2-(3,4-dihydroxyphenyl)-4*H*-chromen-4-one; 5,3′,4′-Trihydroxyflavone, 7,3′,4′-Trihydroxyflavone; and luteolin) is the catechol group in the B-ring [175]. Docking analysis shows three active sites between COX-2 and luteolin with B-ring oriented onto COX-2 hydrophobic pocket (Tyr^385^, Trp^387^, Phe^518^, Ala^201^, Tyr^248^) and luteolin 3,4-dihydroxy groups formed H-bond between Tyr^385^ and Ser^530^ [176]. Regarding on flavonoids and COX-1 inhibitory activity, flavonoids flavone, 5-hydroxyflavone, and 7-hydroxyflavone have none or just one hydroxyl group at B ring. Fewer substitutions in flavonoids backbone is important for COX-1 inhibitory activity because COX-1 pocket is small and the interaction between less substituted flavonoids may occur easier than more substituted molecules [175]. It is reasonable that this inhibitory effect of flavonoids on COX-2 and COX-1 is responsible, at least in part, for their analgesic effect. In addition, because flavonoids are multitarget drugs physiological systems are less affected compared to single target drugs that almost abolish a unique mechanism involved in disease and physiological functions. Because of this, despite the inhibition of COX, flavonoids do not present the common side effects of NSAIDs. On the other hand, flavonoids reduce the side effects caused by NSAIDs. For instance, hypericum perforatum inhibited acetaminophen-induced hepatotoxicity and lethality in mice which is mainly constituted by flavonoids as quercetin and rutin [177,178].

IL-1β is a pro-inflammatory and hyperalgesic cytokine matured by NOD-like receptor (NLR) family, pyrin domain-containing 3 (NLRP3) inflammasome [179]. Lim et al. [180] screened 56 flavonoids towards their SAR in monosodium urate (MSU) crystals-induced IL-1β release by THP-1 cells [180]. Among those, 56 molecules such as flavone, 2′,4′-dihhydroxyflavone, 3′,4′-dichloroflavone, 4′,5,7-trihydroxyflavone (apigenin), 3,4′,5,7-tetrahydroxyflavone (kaempferol), and 3,3′,4′,5,7-pentahydroxyflavone (quercetin) inhibit MSU-induced IL-1β production [180]. Specifically, quercetin interferes with ASC oligomerization, thus, inhibiting inflammasome activation [57], The 4-carbonyl group, 2,3- double bond, and 3-hydroxyl moieties at ring C are important in MSU-induced IL-1β maturation since catechin did not show an inhibitory effect when compared to flavone and quercetin. Also, flavanones naringenin (10 µM) compared to apigenin (10 µM) did not inhibit IL-1β released by MSU [179]. Nevertheless, data in murine bone marrow-derived macrophages show that naringenin (300 µM) reduces the release of IL-1β [181]. Therefore, the lack of activity of naringenin on THP-1 cells might be cell-specific and dependent on concentration and experimental conditions. In addition, 4-hydroxyl substitution at B-ring, such as in apigenin and kaempferol, enhances inhibitory activity of IL-1β production compared to chrysin and galangin. Moreover, hydroxyl substitution at position 4 at B ring combined with 5,7-dihydroxyl at A ring further increase the inhibitory activity in apigenin and kaempferol compared to 4-hydroxyflavone. On the other hand, methylated, methoxylated, and glycosides derivates were unable to inhibit MSU-induced IL-1β release by THP-1 [180]. Thus, the presence of 5,7-dihydroxyl groups at A ring, 4-hydroxyl or 3,4-dichloro substitutions at B ring are important for the inhibition of IL-1β release by MSU. Therefore, flavonoids have important structures that confer their activity such as, 2,3-double-bound bond in conjugation with 4-oxo group, or hydroxyl groups in a specific position as 5,7-dihydroxyl groups at A ring, 4-hydroxyl or 3,4-dichloro substitutions at B ring. The structure of flavonoids is closely related to their activity and changes such as methylation, methoxylations, glycosylation, and polymerization can increase, decrease, or even not alter their activity, such as occur with the *O*-dihydroxy group at the B ring, the 2,3-double-bound bond in conjugation with the 4-oxo group at C ring, and the 3-hydroxyl and 5-hydroxyl groups at the C and A rings. Moreover, flavonoids derivation improves their activity, as in the case of the methoxy substitution in 3,3′,5,5′-Tetramethoxychalcone.

The PI3K/Akt pathway plays an essential role in the regulation of inflammatory responses [182,183,184]. The inhibition of PI3K protein by quercetin and myricetin was investigated ny crystallographic approach. The results demonstrate that the hydrogen bond between the 3′-OH (B ring) of quercetin and the side chain of Lys^833^ mimics the interaction made by the ketone moiety of LY294002 (PI3K inhibitor) and myricetin is recognized through B ring by Val^882^ residue of PI3K [185]. In this sense, the treatment of the T47D cells with epidermal growth factor (EGF) induced Akt phosphorylation at Ser^473^ and pretreatment the cells with quercetin (25 μM) suppressed the EGF-induced Akt phosphorylation at Ser^473^ [56]. These findings provide a molecular rationale for designing molecules based on the inhibition of PI3K/Akt pathway by quercetin and myricetin. More studies are needed to determine flavonoids SAR and their interaction with inflammatory targets aiming to develop flavonoids targeting selected pain and inflammation pathways.

## 4. Clinical Studies and Safety

The use of plants for therapeutic purposes is an ancient practice [186]. As pharmaceutical tools evolve, the discovery of new drugs from plants leads to the isolation of many compounds that are widely used clinically today [186], or originate prototypes for synthesis of new drugs [187,188]. It is estimated that around 48% of new chemical entities discovered between 1981 and 2002 are derived from natural sources, including plant-based ones [189].

Flavonoids represent one of the most studied classes of metabolites with diverse phenolic groups, with an estimated 10,000 different members, having pollinator attractants, antimicrobial, and UV-protective properties in plant kingdom [34]. The antioxidant potential is the most shared among different flavonoids, explaining part of their beneficial effects on human health [34]. As an unprecedented demand for better therapeutic approaches keeps growing worldwide, flavonoids are one of the natural group of compounds that have been extensively studied pre-clinically (Figure 3 and Figure 4) and clinically (Table 1). Randomized controlled trials and other population-based studies suggest that many flavonoids contribute to cardiometabolic health. For instance, kaempferol, naringenin, and hesperetin reduce incidence of cerebrovascular disease, and anthocyanin decreases hypertension in adults [190,191]. Other studies state that anthocyanidins improve disease conditions involving cognitive function, such as Parkinson [192,193]. Incidence of breast, lung, and prostate cancers are affected by consumption of quercetin, myricetin and other flavonols [191,194], as well as other chronic conditions such as asthma and type 2 diabetes [191]. Therefore, in this section we highlight clinical studies addressing the effect of flavonoids or flavonoid-based drugs for the treatment of inflammatory diseases. Table 2 summarizes data discussed in this section.

### 4.1. Hesperidin

Hesperidin (C_28_H_34_O_15_), a flavonoid found in citrus fruits [207], has been linked to the improvement of human endothelial function. That is related to an improve on flow-mediated dilation and lower soluble E-selectin concentrations after 500 mg/day consumption by 24 volunteers, during a 3-week randomized, placebo-controlled, double-blind, crossover trial [195]. In another study, results show a lower diastolic blood pressure and improvement of endothelium-dependent microvascular reactivity in overweight individuals consuming 292 mg of hesperidin during a 4-week randomized, controlled, crossover study. Highe plasma levels of hesperidin correlated with these outcomes [208]. Hesperidin is associated with alterations in lipid profiles as well. A 3-week daily intake of 500 mg hesperidin by 25 individuals with metabolic syndrome reduced total cholesterol and increased HDL levels [209], and 100 mg or 500 mg/d for 6 weeks reduced serum triglyceride levels in hyperlipidemic subjects [210]. Moreover, in a study conducted by Rizza et al. [195], anti-inflammatory effects were observed after 500 mg daily hesperidin treatment, as circulating levels of inflammatory biomarkers (high-sensitivity C-reactive protein and serum amyloid A protein) decreased when compared to placebo-treated patients [195]. In combination with other compounds, hesperidin exerts beneficial properties. Clinical trials with Daflon 500 mg, a venotropic drug composed of diosmin (450 mg) and hesperidin (50 mg), observed vasoprotector effects in subjects suffering from chronic venous insufficiency. That includes improvement of venous capacitance, lessened edema, limitation of skin disorders, stimulation of ulcer healing, and improvements of clinical signs and symptoms (leg pain, heat sensation, heaviness, redness, etc.) [211,212,213]. Moreover, it also reduced the severity, frequency, and duration of the hemorrhoidal attacks. As consequence, patients experience reduced pain, bleeding, and discharge [196,197,198]. Rikkunshito, an herbal medicine of which hesperidin is one of main active ingredients, is used in the treatment of gastrointestinal symptoms of patients with functional dyspepsia [214,215]. In addition, it promotes secretion of ghrelin and, therefore, stimulates appetite in cancer patients suffering from anorexia during chemotherapy [216,217,218]. HMC (C_29_H_36_O_15_), a chalcone methylated compound, derived from hesperidin, with higher solubility, has been described as effective and safe in the treatment of vascular diseases when combined with other compounds such as vitamin C and Ruscus aculeatus extract (Cyclo 3 Fort^®^, Laboratoires Pierre Fabre, Paris, France). Treatment with HMC reduces symptoms such as pain, heaviness, and paresthesia, as reviewed by Boyle et al. [219] and Kakkos and Allaert [220].

### 4.2. Catechins

Catechins are polyphenolic molecules present in various types of teas, commonly related to health benefits due to their antioxidant capacity and attenuation of NFκB activation [221,222,223,224]. Epigallocatechin-3-gallate (EGCG) is the major constituent among catechins present in tea leaves [224]. Regarding safety, an experimental study published in 2016 concluded that EGCG administration presents no toxicity at doses up to 500 mg/kg/day for 13 weeks in rats [225]. As reviewed previously [223,226,227], clinical studies analyzing the effects of green tea consumption linked catechins to cardioprotective and anti-inflammatory effects, such as lower blood pressure, lower levels of serum TNFα, C-reactive protein, cholesterol and triglycerides in patients [199]. Upon green tea consumption, individuals with prostate cancer presented a significant decrease in the disease progression [228,229,230,231] and risk of developing lung cancer was diminished [232,233]. Despite the strong evidence of cancer-preventive effect suggested by some studies, there are conflicting results as well, and several limitations should be considered – genetic variations between individuals and populations, confounding factors, and regarding experimental results, human translational difficulties such as equivalent dose [234]. Furthermore, in a double-blind controlled study, type 2 diabetes patients had improvements in obesity and glucose levels after 582.8 mg or 96.3 mg consumption of catechins for 12 weeks [235]. Bone health improvements and Alzheimer’s disease prevention by catechins in pre-clinical and clinical studies were summarized somewhere else [236,237].

### 4.3. Quercetin

Quercetin (C_15_H_10_O_7_) is the most abundant dietary flavonol [66]. One study published in 2009 aiming to test the effects of 150 mg quercetin/day for 6 weeks in subjects with high-cardiovascular disease risk observed a reduction in systolic blood pressure and plasma oxidized LDL [238]. Another randomized, double-blind, placebo-controlled, crossover study, aiming to test the efficacy of 730 mg quercetin/day for 4 weeks in hypertensive subjects observed reductions in systolic, diastolic and mean arterial pressures [239]. Similar blood-pressure regulation efficacy was observed in type-2 diabetes women taking 500 mg quercetin once daily, for 10 weeks [240]. A meta-analysis of clinical studies evaluating quercetin actions on cardiovascular protection concludes that quercetin supplementation should be considered as an add-on therapeutic approach [241]. Quercetin doses above 500 mg/day show effects on the C-reactive protein levels [242], and in women with polycystic ovary syndrome, 1g quercetin intake for 12 weeks improved adiponectin-mediated insulin resistance and hormonal profiles [242]. Moreover, in 50 rheumatoid arthritis (RA) patients, 500 mg quercetin supplementation during 8 weeks ameliorates clinical symptoms such as early morning stiffness and pain, disease activity, and TNFα plasma levels [200].

### 4.4. Apigenin

Apigenin (C_15_H_10_O_5_) consumption is considered safe, although muscle relaxation and sedation can occur when 30–100 mg/kg body weight doses are administrated [243]. Anxiolytic effects were observed in patients with generalized anxiety disorder treated with chamomile extract for 8 weeks [244]. A crossover double-blind clinical trial, enrolling 100 patients diagnosed with migraine without aura, tested the efficacy of an oleogel with chamomile extracts. Results showed that pain, nausea, vomiting, photophobia, and phonophobia significantly decreased [201]. Topic application of apigenin-containing cream in 20 women reduces skin aging, besides increasing dermal density and elasticity [245]. In addition, treatment with a flavonoid mixture composed of 20 mg epigallocathechin-gallate plus 20 mg apigenin for 2–5 years reduced the recurrence rate of colon neoplasia in patients with resected colon cancer [246].

### 4.5. Flavonoid-Based Compounds

Plant extracts containing different classes of flavonoids have been studied in clinical trials and presents promising results. Silymarin (Livergol^®^, Goldaruo pharmaceutical, Iran), present in species derived from Silybum marianum, contains seven flavolignans and the flavonoid taxifolin (C_15_H_12_O_7_) [247]. Parameters such as joint swelling, tenderness, and pain were reduced in RA patients taking 420 mg of silymarin daily for three months [202]. A meta-analysis involving 8 randomized controlled trials found that Silymarin intake has efficacy in the treatment of alcoholic fatty disease by reducing transaminases levels in patients [248]. Pycnogenol^®^ (Horphag Research Ltd., UK, Geneve, Switzerland), extract of *Pinus maritima*, is a mixture of flavonoids, mainly procyanidins [249]. Sixty-seven subjects with osteoarthritis (OA) taking 220 mg of Pycnogenol^®^ daily for 3 weeks presented a decrease in C reactive protein levels and reduced use of painkillers and non-steroidal anti-inflammatory drugs [203,250]. In other trials with OA patients taking 150 mg/day Pycnogenol^®^, relief from daily pain, stiffness, and physical function were observed [204,205]. Alvocidib, or Flavopiridol (Tolero Pharmaceuticals, Inc.) is a synthetic analog of a naturally occurring flavone extracted from Dysoxylum binectariferum. It has a CDK9 kinase inhibitor activity, arresting cell cycle at G1 phase, and, therefore, has been shown as a promising therapy for patients with acute myeloid leukemia and chronic lymphocytic leukemia [206,251]. Enzogenol^®^, a flavonoid-rich extract of Pinus radiate containing approximately 80% total proanthocyanidins and other water-soluble flavonoids, has been linked to neuroprotection properties [252,253] and beneficial vascular effects [254]. Furthermore, in a double-blind, placebo-controlled trial, women taking Colladeen^®^ (Lamberts Healthcare Limited, UK) – 320 mg oligomeric procyanidins – improves leg health and reduces fluid retention in pre-menopausal phase [255].

So far, most clinical reports in the literature states flavonoids consumption as safe. Despite the main source of flavonoids is diet, it is important to highlight that supplement intake is growing, which leads to the concern of high-dosage toxicity and/or interactions with other dietary elements or medications, resulting in possible adverse effects [256]. Although there are a lot of patent requested to pharmaceutical compositions containing flavonoids and to use of flavonoids [257], to our knowledge, the Food and Drug Administration (FDA) has not yet endorsed any flavonoids as pharmaceutical drugs. Although antioxidant properties are commonly linked to flavonoid molecules, under specific conditions they can be prooxidant (hydroxyl radicals can be produced due to their iron and copper reducing activities [258]) and present side effects, as genotoxicity, nausea, and headache [259,260]. Supplementation of high doses may cause antithyroid and goitrogenic effects, in addition to lower bioavailability of vitamin C, trace elements, and vitamins such as folate [256]. While presenting safe pre-clinical and clinical profiles, further studies addressing toxicity, interactions, contraindications, and safety of flavonoids intake are needed to avoid indiscriminate consumption based on the idea that “natural molecules” do no harm.

## 5. Development of Pharmaceutical Formulations Containing Flavonoids

Different types of formulations have been applied to flavonoids, such as topical and oral formulations [261,262]. Topical formulations of flavonoids are a promising therapeutic option to provide a site-specific application of the drug with important pharmaceutical uses [261]. Several studies demonstrated that topical formulations containing quercetin, trans-chalcone, naringenin, and hesperidin methyl chalcone inhibit the UVB irradiation-induced inflammation and stress oxidative [50,61,72,263]. Therefore, their topical use may provide the required photochemical protection in addition to human sunscreens. Quercetin gel reduced CFA-induced inflammation such as increased paw edema, erythema, swelling, joint stiffness and disturb in the movement [264]. In this regard, treatments with flavonoids incorporated into topical formulations can decrease the local inflammatory process, pain, and stress oxidative.

The most challenging factor is the flavonoids’ low water solubility, which leads to lower absorption and consequently lower bioavailability after oral administration [265]. In this sense, protective delivery systems of flavonoids may be a promising therapeutic option to significantly expand the water solubility, dissolution, absorption, thermal stability and bioavailability of flavonoid, thus the construction of flavonoid microcapsules, nanoparticles and nano-formulations is an effective approach to increase its bioavailability, including the use of liposomes, inclusion complex (cyclodextrin and phospholipid complexes), micelles, and solid dispersion (Table 3).

Microencapsulation (pectin/casein by complex coacervation) of quercetin ameliorates acetic acid-induced colitis by providing a controlled release of quercetin at the mouse colon and improves the anti-inflammatory and antioxidant effects of quercetin compared to the non-encapsulated drug [69]. Rutin-Loaded Microparticles (pectin/casein by complex coacervation as well) induce the inhibition of carrageenan-induced mechanical hyperalgesia better than nonmicroencapsulated rutin does [266].

Superparamagnetic iron oxide nanoparticle (SPION) drug delivery system enhanced the bioavailability of quercetin. The SPION increases blood circulation time of quercetin and increases the concentration of quercetin in the brain that leads to higher antioxidant activity and more efficient interactions of quercetin with RSK2, MSK1, CytC, Cdc42, Apaf1, FADD, CRK proteins [267]. Naringenin nanosuspensions formulated using polyvinylpyrrolidone displayed a higher dissolution amount (91 ± 4.4% during 60 min) compared to pure naringenin (42 ± 0.41%). The apparent and effective permeability of naringenin nanosuspension was increased as compared to the pure naringenin. The in vivo naringenin nanosuspension treatment showed maximum concentration and area under curve (0–24 h) values approximately 2-fold superior than the pure drug [268]. The flavonoid fisetin–loaded polymeric nanoparticles had protected and preserved the release of flavonoid fisetin in gastric and intestinal conditions. The ABTS scavenging capacity of flavonoid fisetin, as well as α-glucosidase inhibition activity, were enhanced about 20-fold compared to pure compounds [269]. The nanoparticles and microencapsulates provide controlled release of agent and can be further optimized to be used as an efficient flavonoids’ delivery.

Self-Nanoemulsifying Drug Delivery System (SNEDDS) has been proved to improve the bioavailability of naringenin, quercetin, and rutin. The drug concentration time-curve of naringenin from SNEDDS revealed a significant increase in naringenin absorption compared to naringenin suspension [270]. The optimized SNEDDS formulation of rutin had a globule size in the nanometric range, which led to faster and better absorption in comparison to rutin suspension [272,273]. The quercetin SNEDDS significantly improved quercetin transport across a human colon cell monolayer and demonstrated rapid absorption within 40 min of oral ingestion [271]. SNEDDS increased absorption, optimum globule size and higher solubility as well as higher bioavailability. Thus, the SNEDDS could be used an effective approach for enhancing the solubility and bioavailability of flavonoids.

The liposomal encapsulation for four types of flavonoids demonstrated that the quercetin-loaded liposomes showed higher stability and more antioxidant capacity than those loading rutin, luteolin and kaempferol [274,275]. The water solubility of quercetin and rutin was improved by phospholipid complex [276,277]. There was no statistical difference between the quercetin or rutin complex and quercetin or rutin in the in vitro antioxidant activity, indicating that the process of complexation does not adversely affect the bioactivity of the active ingredient [276,277]. Naringenin-cyclodextrin complex presented a release of 98.0 - 100% at 60 min and enhanced the thermal stability of naringenin [278]. Polymeric micelles exhibited sustained release of quercetin or rutin compared to free quercetin or rutin, as demonstrated by in vitro assays. The solubility of quercetin and rutin was markedly improved compared to pure molecules [279,280]. Amorphous solid dispersion (ASD) of naringenin has been developed with various polymers like cellulose derivatives. Poly-vinylpyrrolidinone-based ASDs demonstrated drug release in gastric (1.2 pH buffer) and small intestine (6.8 pH buffer) conditions. The cellulosic polymer delivers naringenin selectively at a neutral pH at the site for its absorption, whereas it inhibits the drug degradation in the gastric environment environment [281]. At present, a wide range of protective drug delivery systems are available, showing promising results for flavonoids’ delivery. In this sense, protective formulation strategies can be explored as per the pharmacological activity of flavonoids.

## 6. Conclusions

We have reviewed pre-clinical and clinical data about the anti-inflammatory and analgesic properties of flavonoids. Most studies have been demonstrating the potential therapeutic role and efficacy of flavonoids in cardiovascular diseases, osteoarthritis, Parkinson disease, colitis, cancer pain, arthritis, and neuropathic pain. The mechanisms of action of flavonoids are yet to be fully elucidated, but many studies have shown their relevance as a potent anti-inflammatory, analgesic, and antioxidative group of molecules. Indeed, flavonoids can block the expression and activation of many cellular regulatory proteins such as cytokines and transcription factors, resulting in diminished cellular inflammatory responses and pain. In conclusion, in view of the pharmacological activities of flavonoids, it could also be interesting to further develop protective delivery formulations containing flavonoids to treat inflammatory diseases and pain, since promising effects were already observed [69,266].

Flavonoids are multi-target molecules, and increasing attention has been given to these molecules due to their anti-inflammatory and analgesic properties. Diminishing the activity of varied pathways seems to present fewer side effects than abolishing the activity of one target, since, in general, the targets also have endogenous physiological roles. Flavonoids block the synthesis of inflammatory mediators such as IL-1β, TNF-α, NO, and COX-2, suppress VEGF and ICAM-1 expression, along with the activation of STAT3, NFkB, NLRP3 inflammasome, and MAP kinases pathways. Regardless of their broad pharmacological properties, flavonoids show a poor water solubility, inadequate permeability, and constrained bioavailability, potentially requiring high doses to show efficacy in [282]. Phase 2 metabolism is known to affect the bioavailability of flavonoids in humans [283]. Generally, most flavonoids experience sulfation, methylation, and glucuronidation in the small digestive system and liver and conjugated metabolites can be found in plasma after flavonoid ingestion [284]. Nevertheless, some metabolites of flavonoids are still active [285]. In such manner, various endeavors have been made to expand bioavailability, for example, improving the intestinal absorption by means of utilization of absorption enhancers, novel delivery systems; improving metabolic stability; changing the site of absorption from large intestine to small intestine.

Thus, flavonoid pharmacology, therapeutics, and pharmaceutical development remain a hot topic in inflammatory diseases and pain treatment.

## Figures and Tables

**Table 1:** Pre-clinical studies analyzing the effects of different flavonoids on cell lines.

Flavonoids Groups	Flavonoid	Cell Line		Effects	Refs
**Flavonols**	Quercetin	macrophages	RAW 264.7	Reduce TNF-α, IL-1β and IL-6 production	[75]
			BMDM	Inhibit ASC speck formation and ASC oligomerization	[57]
			BMDM	Modulate M1 and M2	[85]
			RAW 264.7		[83]
			J 774		[84]
		neutrophils	Human neutrophils	Modulate actin polymerization	[76]
		dendritic cell	BMDC	Activation and Maturation	[81,82]
		mast cells	HMC-1	Reduce TNF-α, IL-1β, IL-8 and IL-6 production	[77]
			hCBMCs	Reduce histamine, leukotrienes and PGD2	[78]
		monocytes	Human THP-1 monocytic cells THP-1	Reduce TNF-α, and IL-1β production	[79,80]
	Rutin	macrophages	RAW 264.7	promote M2 polarization	[95]
			CD11b+ primary macrophages		
		neutrophils	Human peripheral blood neutrophils	Reduce NO and TNF-*α* production	[96]
		mast cells	HMC-1	Reduce TNF-α, IL-1β, IL-8 and IL-6 production	[77]
		monocytes	Human THP-1	Inhibit adhesion	[97]
**Flavones**	Apigenin	macrophages	ANA-1	Modulate macrophages polarization	[102]
			RAW264.7		
			RAW 264.7	Reduce NO production and COX-2 expression	[105]
		neutrophils	Human peripheral blood neutrophils	Down-regulation of Mcl-1	[103]
		dendritic cell	BMDC	Inhibit maturation and migration	[35,104]
		mast cells	HMC-1	Inhibit TNF-α, IL-8, IL-6, GM-CSF, and COX-2 expression and NF-kB activation	[106]
		monocytes	monocytes to HUVEC	Reduce TNF-α, production	[105]
	Vitexin	macrophages	RAW 264.7	Inhibit TNF-α, IL-1β, NO, PGE2 and increase in IL-10 release	[113]
		neutrophils	Human peripheral blood neutrophils	Reduce NO, TNF-α, and MPO production	[112]
		mast cells	RBL-2H3	Prevent degranulation	[111]
	Diosmin	macrophages	RAW264.7	Reduce NO, PGE2, IL-6, IL-12, TNF-α production	[120]
**Flavanones**	Naringenin	macrophages	U937	Regulate activation	[129]
		neutrophils	Human peripheral blood neutrophils	Regulate microbicidal activity	[130]
		dendritic cell	BMDC	Reduce maturation	[131]
	Hesperidin	macrophages	RAW264.7	Modulate M1 polarization	[70]
		neutrophils	Human peripheral blood neutrophils	Reduce generate superoxide radical	[140]
		mast cells	HMC-1	Reduce TNF-α and IL-1β production	[141]
**Chalcone**	Trans-chalcone	macrophages	BMDM	Reduce IL-1β production	[42]
	Hesperidin methyl chalcone	macrophages	RAW264.7	Reduce IL-33, TNF-α, and IL-6 levels	[141]
**Flavan-3-ols**	Epigallocatechin-3-gallate	macrophages	RAW 264.7	Reduce NO, prostaglandin PGE2 and COX-2 production	[147]
		neutrophils	Murine peritoneal neutrophils	Reduce chemotaxis	[150]
		dendritic cell	Human dendritic cell	Differentiation and maturation	[148]
		mast cells	RBL-2H3	Inhibit degranulation	[149]

BMDM: Bone Marrow-derived Macrophage; BMDC: Bone marrow-derived dendritic cells; hCBMCs: Human umbilical cord blood-derived mast cells.

**Table 2:** Clinical studies analyzing the effects of different flavonoids in diets or as supplements in patients.

Flavonoid/Flavonoid-Based Compound	*n*	Treatment	Duration	Outcomes	Refs
**Flavanones, anthocyanins, flavan-3-ols, flavonols, flavones, and polymers**	49,281 men in the HPFS and 80,336 women from the NSH	Food frequency questionnaire	20–22 years of follow-up	Intake of some flavonoids may reduce Parkinson disease risk, particularly in men	[193]
**Hesperidin**	24	500 mg, daily	3 weeks	Increased flow-mediated dilation and reduced concentrations of circulating inflammatory biomarkers	[195]
	100	Daflon 500 mg (3 tablets bid. the first 4 days and 2 tablets bid. the following 3 days)	7 days	Clinical severity, inflammation, congestion, edema, prolapse, duration, and severity of hemorrhoidal episode diminished	[196]
	120	Daflon 500 mg, 2 tablets, daily	2 months	The overall symptom score decreased when compared to placebo	[197]
	105	Daflon 500 mg, 2 tablets, daily	4 weeks + follow-up for 6 months	Improvement in pain, heaviness, bleeding, pruritus, and mucosal discharge from baseline	[198]
	56	379 mg of green tea extract	3 months	Improvements in blood pressure, insulin resistance, inflammation and oxidative stress, and lipid profile in patients with obesity-related hypertension	[199]
**Quercetin**	50	500 mg, daily	8 weeks	Improvements in clinical symptoms, disease activity, hs-TNFα, and health assessment questionnaire in women with RA	[200]
**Apigenin**	100	2 mL of an oleogel preparation of reformulated traditional chamomile oil	Topical application, once	Pain, nausea, vomiting, photophobia, and phonophobia significantly decreased in patients with migraine without aura	[201]
**Silymarin (Livergol^®^, Goldaruo pharmaceutical, Iran)**	44	420 mg, daily	90 days	Joint swelling, tenderness, and pain were reduced	[202]
**Pycnogenol^®^ (Horphag Research Ltd., UK, Geneve, Switzerland)**	67	220 mg, daily	3 weeks	Patients with OA decreased C reactive protein levels and reduced use of painkillers and non-steroidal anti-inflammatory drugs	[203]
	100	150 mg/day	3 months	Patients with OA presented relief from daily pain, stiffness, and physical function	[204]
	37	150 mg/day	3 months	Alleviating OA symptoms and reducing the need for NSAIDs or COX-2 inhibitors administration	[205]
**Alvocidib or Flavopiridol (Tolero Pharmaceuticals, Inc.)**	10	30-min loading dose of 30 mg/m (2) followed by a 4-h infusion of 30 mg/m (2) once weekly	3 weeks every 5 weeks, twice	Reduction in tumor burden on chronic lymphocytic leukemia patients	[206]

NHS: Nurses’ Health Study; HPFS: Health Professionals Follow-Up Study; hs-TNFα: high-sensitivity tumor necrosis factor-α; RA: rheumatoid arthritis; OA: osteoarthritis.

**Table 3:** Delivery systems containing flavonoids and key improvement effects.

Formulations Containing Flavonoids	Flavonoids	Biological Effects
**Microparticles**	Quercetin [69] Rutin [266]	↑ bioavailability ↑ absorption ↑ solubility ↑ stability ↑ efficacy
**Nanoparticles**	Quercetin [267] Naringenin [268] Fisetin [269]	
**SNEDDS**	Naringenin [270] Quercetin [271] Rutin [272,273]	
**Liposomal**	Quercetin [274,275] Rutin [274] Kaempferol [275] Luteolin [275]	
**Inclusion Complex**	Quercetin [276] Rutin [277] Naringenin [278]	
**Micelles**	Quercetin [279] Rutin [280]	
**Solid Dispersion**	Naringenin [281]	

Pre-clinical findings.
